# Social determinants of health in India: progress and inequities across states

**DOI:** 10.1186/s12939-014-0088-0

**Published:** 2014-10-08

**Authors:** Krycia Cowling, Rakhi Dandona, Lalit Dandona

**Affiliations:** Public Health Foundation of India, Plot 47, Sector 44, Gurgaon, 122002 New Delhi India; Institute for Health Metrics and Evaluation, University of Washington, Seattle, Washington USA

**Keywords:** Health inequalities, India, Multidimensional poverty, Social determinants of health, Subnational estimation

## Abstract

**Introduction:**

Despite the recognized importance of social determinants of health (SDH) in India, no compilation of the status of and inequities in SDH across India has been published. To address this gap, we assessed the levels and trends in major SDH in India from 1990 onwards and explored inequities by state, gender, caste, and urbanicity.

**Methods:**

Household- and individual-level SDH indicators were extracted from national household surveys conducted between 1990 and 2011 and means were computed across population subgroups and over time. The multidimensional poverty index (MPI), a composite measure of health, education, and standard of living, was calculated for all three rounds of the National Family Health Survey, adjusting the methodology to generate comparable findings from the three datasets. Data from government agencies were analyzed to assess voting patterns, political participation, and air and water pollution.

**Results:**

Changes in the MPI demonstrate progress in each domain over time, but high rates persist in important areas: the majority of households in India use indoor biomass fuel and have unimproved sanitation, and over one-third of households with a child under the age of 3 years have undernourished children. There are large, but narrowing, gender gaps in education indicators, but no measurable change in women’s participation in governance or the labor force. Less than 25% of workers have job security and fewer than 15% have any social security benefit. Alarming rates of air pollution are observed, with particulate matter concentrations persistently above the critical level at over 50% of monitoring stations.

**Conclusions:**

This assessment indicates that air pollution (indoor and outdoor), child undernutrition, unimproved sanitation, employment conditions, and gender inequality are priority areas for public policy related to SDH in India.

**Electronic supplementary material:**

The online version of this article (doi:10.1186/s12939-014-0088-0) contains supplementary material, which is available to authorized users.

## Introduction

The 2008 publication of the Commission on Social Determinants of Health (CSDH) report focused attention on the crucial role of living conditions for preventing morbidity and mortality, improving health status, and dictating inequalities in health outcomes and utilization of health services [1]. One of three overarching recommendations of the report was to “measure the problem, evaluate action, [and] expand the knowledge base” [1]. However, since the report’s publication, a regional-level assessment for the European region is published [2], but detailed assessments of social determinants of health (SDH) at the country level are not available. As the relative importance of different social determinants of health, the availability of data for tracking progress, and the history of relevant government actions vary by country, it is important to extend this discussion to the country level for national policy relevance.

To guide this review, we used the CSDH framework, which defines SDH as the impacts of conditions “in which people are born, grow, live, work, and age” on health status. Inequalities are inextricably linked to this framework as it is often socially constructed inequities in access and exposure to key determinants that make them significant for health. A large and growing literature links these determinants to health outcomes; these determinants vary from more upstream factors, such as inclusion in political processes, to more downstream factors, such as access to clean water and sanitation [3,4]. Studies finding associations between SDH and health are further strengthened by research demonstrating causality, such as recent reports finding improved flooring led to decreases in the incidence of childhood infections [5] and that households given stoves producing less indoor air pollution had less severe pneumonia cases [6]. The broad definition of SDH employed by the CSDH encompasses a web of factors that interact in multiple and complex ways; as a result most analyses are not able to include all underlying factors or do a full analysis of the ways determinants affect one another. With this limitation, this review includes major SDH in India for which there are available data, examined for important inequities when possible. Key SDH not included in this review due to the absence of data are described in the discussion.

The recent High Level Expert Group Report on Universal Health Coverage for India discussed the importance of synergistic action on SDH to ongoing efforts to achieve universal health coverage [7]. As a basis for identifying and setting SDH priorities in India, there is no compilation of the levels, trends, and inequities in these indicators. Inequalities by location are common in all countries; in India, where many states’ populations are equivalent to those of the world’s most populous countries, this is especially significant. In this report, we focus on subnational variations, presenting analyses of the available data for measuring key SDH in India over the past two decades. We assess inequities by geography, caste, and gender, and identify priorities for public policy.

## Methods

We conducted secondary analyses of publicly available data from nationally representative household surveys and various national government agencies. All data sources used in this analysis are listed in Table 1.

**Table 1:** **National data sources referred to in this study for measuring the social determinants of health in India, 1990-2013**

**Data source**	**Years**	**SDH topics**
National Family Health Survey	1992-93	Living conditions, nutritional status, education
	1998-2000	
	2005-06	
National Sample Survey Organization: Employment Schedule	1993-94	Employment, education
	1999-2000	
	2004-05	
	2009-10	
District Level Household and Facility Survey	1998-99	Living conditions, education
	2002-04	
	2007-09	
Annual Health Survey *(less developed states only)*	2010-11	Living conditions, education
HUNGaMA (Hunger and Malnutrition) Survey	2011	Undernutrition
Election Commission of India	1990-2013	Characteristics of candidates & voters in state and national elections
Central Pollution Control Board	1995-2011	Air quality, water quality
Planning Commission	1990-2013	Poverty
United Nations Statistics Division: Indicators on women and men	varied	Gender inequality

### Computation of multidimensional poverty index

In this study, we utilize the Multidimensional Poverty Index (MPI) to portray a more nuanced measurement of poverty than strictly income-based measures and to provide a composite indicator of several social determinants of health. We computed the MPI by adapting existing methodology [8]. This index weights ten indicators across three dimensions: health – 1) *any child death in the household, 2) any child malnourished in the household*; education – 3) *no household member with five or more years of education, 4) any school-age child not attending school*; and standard of living – 5) *unimproved source of drinking water, 6) unimproved sanitation, 7) indoor biomass fuel use, 8) low quality housing, 9) lack of electricity, and 10) limited household asset ownership* (Table 2) [8,9]. We utilized three rounds of National Family Health Surveys (NFHS) conducted in 1992-93, 1998-2000, and 2005-06 to compute the MPI [10-12]. Adaptations to the methodology were required to utilize all three datasets, but these changes preserve the significant features of the MPI (described in detail in Additional file 1: “Adaptions to the MPI methodology”). The priority for our methodology was to generate comparable results across the three time periods, as all three datasets have not been previously incorporated in an assessment of the MPI at the state level in India [13-15].

**Table 2:** **Ten indicators across three dimensions that comprise the Multidimensional Poverty Index (MPI)** [8]

**Dimension**	**Indicator**	**Definition**
Health	Child death	One or more children born in the household in the last five years have died
	Child undernutrition	At least one child in the household under the age of 3 is underweight
Education	Adult education	No household member has completed five or more years of schooling
	Child not in school	At least one school-aged child is not enrolled in school
Standard of living	Unimproved water	The drinking water source does not meet the WHO/UNICEF JMP criteria for “improved”
	Unimproved sanitation	The sanitation facilities do not meet the WHO/UNICEF JMP criteria for “improved”
	Indoor biomass fuel use	The household cooks food with biomass fuels, as defined in the MDGs
	Low quality housing	The main housing material is kachha or semi-pucca
	No electricity	The household has no electricity
	Limited assets	The household has no car or truck and owns at most one of these - bicycle, motorbike, radio, refrigerator, or television

Definitions presented here are those used in this analysis. The original MPI indicators and detailed descriptions of the rationale and methodology for each adaptation are included in Additional file 1: “Adaptions to the MPI methodology”.

#### Deprivation at household level

Following established methodology, we computed the MPI headcount ratio for each NFHS round and subpopulations defined by state, caste, and urbanicity [8]. Each household’s MPI score was a weighted average of deprivations across all indicators; 0 indicating no deprivation and 1 indicating complete deprivation. Any household with a score greater than 0.33 was identified as poor, and the headcount ratio was calculated as the total number of household members in poor households divided by the total household members in the population.

To separately explore the ten indicators of the MPI, we computed the proportion of eligible households deprived in each. All households had the necessary data for seven of the ten indicators; however, only households with a birth in the last five years, a living child under the age of 3 years, and a child aged 7 to 14 years were eligible for computation of the *child death, child underweight,* and *child not in school* indicators, respectively.

##### Detailed assessment of SDH within MPI

The most recent dataset usable for computing the MPI was the 2005-06 NFHS. For a more current assessment of SDH indicators within the MPI, we computed these same metrics from newer surveys, when available. We used the 2007-09 District Level Household and Facility Survey (DLHS) [16] to assess the proportion of households deprived in multiple indicators of the standard of living domain, and the 2009-10 round of the National Sample Survey Organization (NSSO) Employment Survey for updated estimates of education indicators [17].

### Other SDH

#### Employment and financial protection

We used four rounds of NSSO Employment Surveys conducted from 1993-94 to 2009-10 to assess employment-related SDH [18]. We computed the proportion of households with any household member receiving any employment-based financial protection. Among the employed population, we assessed the proportion working in several major industry categories. Finally, we assessed the proportion of men and women aged 15-59 years and children aged 5-14 years who reported working or looking for work as their primary activity.

#### Political participation

We utilized election and voting data from the Election Commission of India [19,20]. We calculated the proportion of candidates by gender and caste from data on candidates for state elections between 2005 and 2012. We used voting data from state elections between 1990 and 2013 to assess voter participation by gender and caste.

#### Environment

Air and water quality monitoring data were compiled from the Central Pollution Control Board (CPCB) [21,22]. (Additional data provided by CPCB via personal communication). Using data from 2000 to 2010 for over 350 air quality monitoring stations nationwide, we computed the proportion of stations with pollutant levels above set thresholds. Nitrogen dioxide (NO_2_), sulfur dioxide (SO_2_), and particulate matter less than 10 micrograms in diameter (PM_10_) are monitored for compliance with National Ambient Air Quality Standards (NAAQS) [23]. These thresholds for SO_2_ and PM_10_ match interim targets in World Health Organization guidelines, and lower levels are advisable [24].

Water quality is also measured nationwide; there were more than 2500 monitoring stations as of 2011 [23], the majority in rivers and the remainder in other water bodies. The CPCB has set targets for levels of biochemical oxygen demand (B.O.D.), total coliform concentration, and fecal coliform concentration in each type of water source. We assessed data by station from 2011 to identify the proportion of monitoring locations with concentrations exceeding these thresholds.

### State groups used for analysis

We present analyses at the state level for the 35 states and union territories in India by classifying these into two groups – less developed and more developed states. The less developed states include the Empowered Action Group (EAG) states: Bihar, Chhattisgarh, Jharkhand, Madhya Pradesh, Orissa, Rajasthan, Uttar Pradesh, and Uttarakhand [25], and the northeast states of Arunachal Pradesh, Assam, Manipur, Meghalaya, Mizoram, Nagaland, Sikkim, and Tripura. The remaining 19 states and union territories comprise the more developed states. Andhra Pradesh was split into two states in 2014; this analysis was conducted before this division.

For each indicator, we computed means at the state level as well as for subpopulations defined by urbanicity and caste. Households were classified as either urban or rural, and caste was defined as scheduled caste/scheduled tribe (SC/ST) or other, based on the relevant variables available in each dataset. We present gender-based analyses where relevant. All analyses were conducted using Stata versions 12 and 13.

## Results

### Multidimensional poverty index

**Figure 1:**
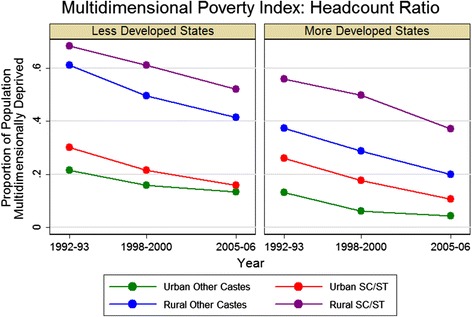
**Multidimensional Poverty Index (MPI) headcount ratio during three rounds of the National Family Health Survey.** The headcount ratio is defined as the proportion of the population living in a household which has a weighted deprivation score greater than 0.33 (on a scale of 0 to 1 with 0 being not deprived in any component and 1 being deprived in all ten components), across the dimensions of standard of living, education, and health. SC/ST is Scheduled Caste or Scheduled Tribe; Other is all other castes.

**Figure 2:**
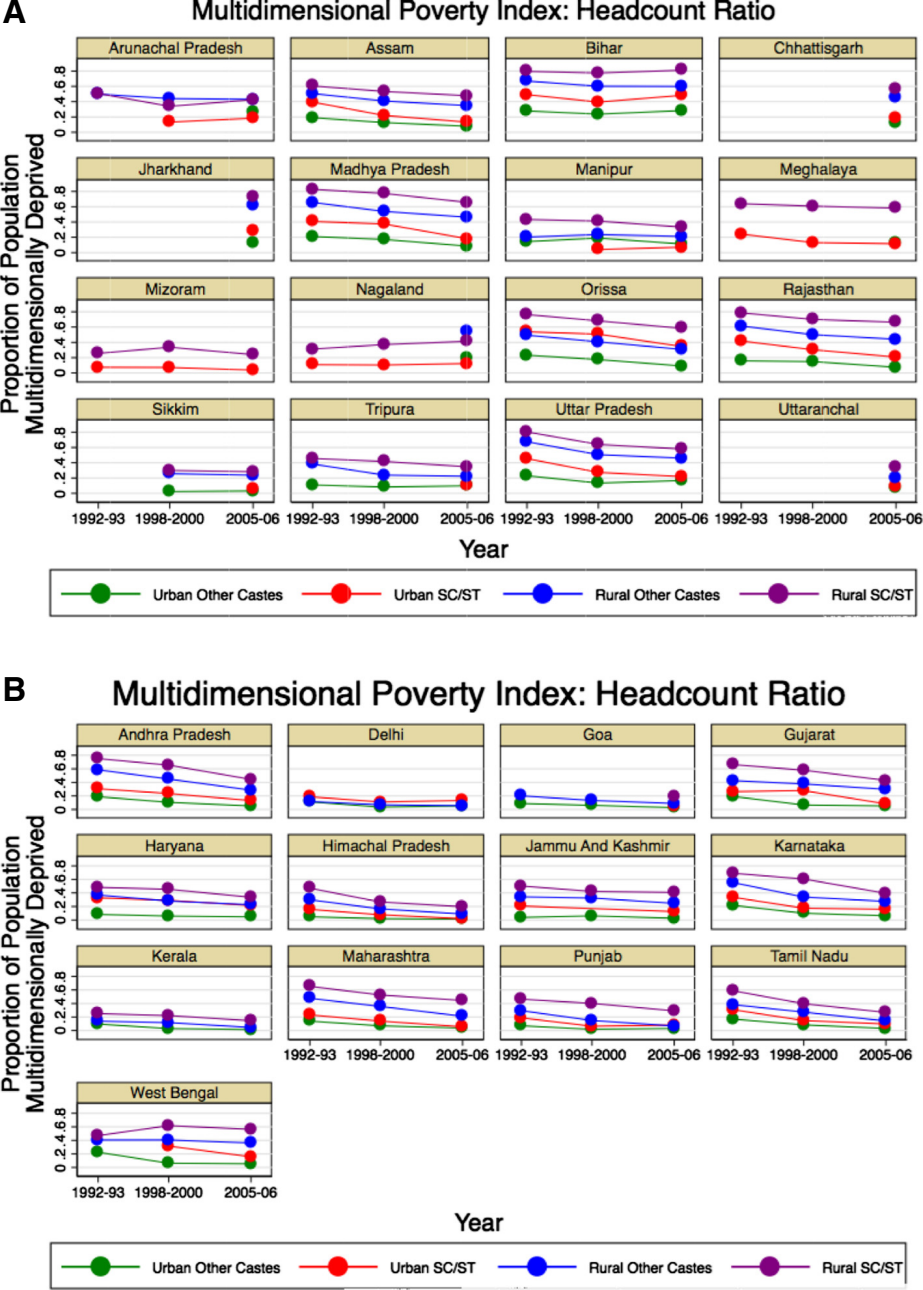
**Multidimensional Poverty Index Headcount Ratio: state-level trends.** NFHS-1, 2, and 3, disaggregated by urban/rural location and caste, for **A)** less developed states and **B)** more developed states. Results are not presented for subpopulations (defined by survey year, state, urbanicity, and caste) if the sample size was less than 100 households.

The MPI headcount ratio in each survey-year for states grouped by level of development and disaggregated by urban/rural location and caste are presented in Figure 1. Results by state are presented in Figure 2. Using this index, 42%, 35.7%, and 24.7% of the Indian population were identified as multidimensionally poor in 1992-93, 1998-2000, and 2005-06, respectively. In comparison, the national poverty headcount ratio using the Government of India’s income-based approach showed much less of a decline from 1993-94 to 2004-05: from 45.3% to 37.2% [26]. Significant disparities in the MPI exist across subgroups of the population. Households in less developed states were more deprived in each round of the survey overall, as well as within each subpopulation defined by caste and urbanicity, compared to households in more developed states. Across both state groups, greater disparities are observable between urban and rural households than between castes: in 2005-06, the headcount ratio gap between urban and rural households was 20 to 30% versus 10 to 15% for SC/ST and other households. However, there was a larger reduction in multidimensional poverty in rural subgroups; the headcount ratio among rural households declined by over 15% in both groups of states between 1992-93 and 2005-06 as compared with a less than 10% decline in urban households.

#### Deprivation at household level

**Figure 3:**
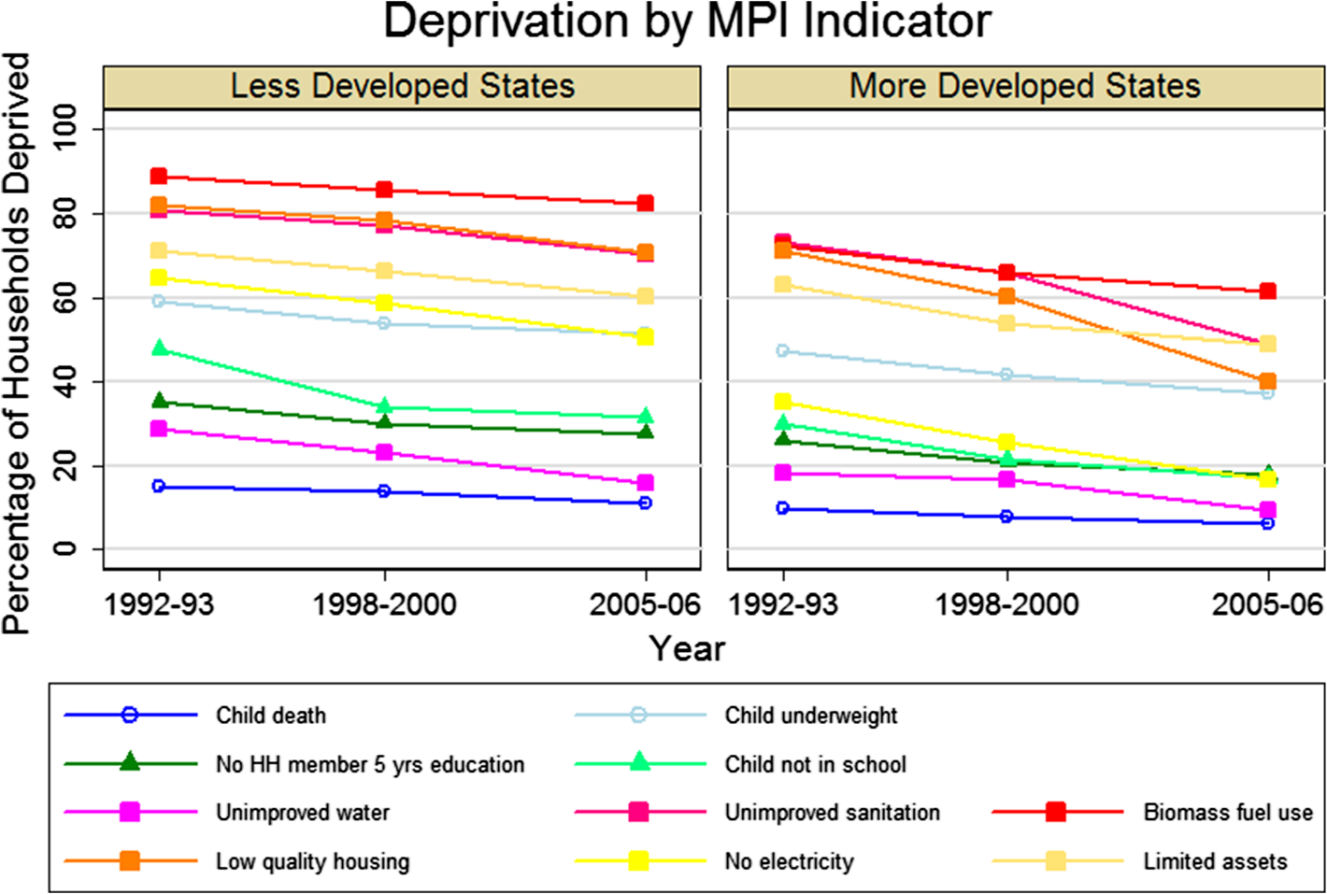
**Change over time in the percentage of eligible households deprived in each of the components of the Multidimensional Poverty Index (MPI).** Symbols indicate the dimension of each indicator (circle for health, triangle for education, and square for standard of living).

Figure 3 presents the proportion of households deprived in each of the ten MPI indicators over time. Deprivations in the standard of living dimension were the most prevalent across all survey periods. Indoor biomass fuel use and unimproved sanitation had the highest proportions of deprivation across both groups of states; rates of low quality housing and limited asset ownership were also high, especially in the less developed states. The most marked progress was in the reduction of unimproved sanitation and low quality housing between 1998-2000 and 2005-06 in the more developed states. The disparity between less developed and more developed states was largest for lack of electricity - 50.4% versus 16.7% of households deprived in 2005-06.

Among households with available data to estimate all of the indicators comprising the MPI, correlations between these various indicators were low across dimensions. The lowest observed correlations were for each of the health outcome indicators with *unimproved source of drinking water:* Pearson’s coefficient with *child death* = 0.03 and Pearson’s coefficient with *child malnourished* = 0.04. The highest correlations were observed intra-dimensionally, for the indicators within the education dimension (Pearson’s coefficient = 0.36) and the standard of living dimension (maximum Pearson’s coefficient = 0.56). This suggests that the index accurately captures various aspects of multi-dimensional poverty and that indicators within the dimensions are appropriately grouped. The role of these multidimensional poverty indicators as social determinants of health is supported by household-level logistic regressions using the health outcome indicators (*child death* and *child malnourished*) as functions of the other indicators. In these models, all relationships were in the direction expected and almost all predictors were statistically significant. In both models, the largest coefficient was for *unimproved sanitation*; effect sizes were generally larger in the model predicting *child malnourished.* These and other analyses of the indicators within and across the domains of the MPI, as well as correlations of the MPI headcount ratio with other state-level SDH, are presented in Additional file 2: “Correlations and regression with SDH indicators.”

#### Indoor biomass fuel use and unimproved sanitation

**Figure 4:**
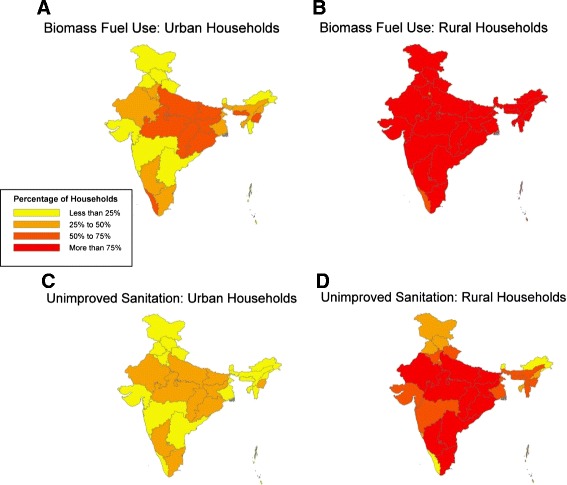
**Interstate variations in the proportion of households using biomass fuel and with unimproved sanitation.** Biomass fuel use in **A)** urban and **B)** rural areas and the proportion of households with unimproved sanitation in **C)** urban and **D)** rural areas. Data are from the 2007-09 District Level Household Survey; as data for Nagaland are not available from this survey, data from the 2005-06 National Family Health Survey are used for this state.

Figure 4 displays the proportion of households in each state using indoor biomass fuel and with unimproved sanitation, by urban/rural location, from the 2007-09 DLHS. Rates of biomass fuel use in rural households were high across India, with more than three-quarters of rural households in all but seven states using biomass fuel. In urban areas, use is concentrated in the less developed states.

Rates of unimproved sanitation are also alarmingly high in rural areas. Only five states have achieved less than one-quarter of rural households with unimproved sanitation. In all of the EAG states except Uttarakhand, as well as in Karnataka, Tamil Nadu, and Manipur, 25% to 50% of urban households also have unimproved sanitation.

#### Child undernutrition

**Figure 5:**
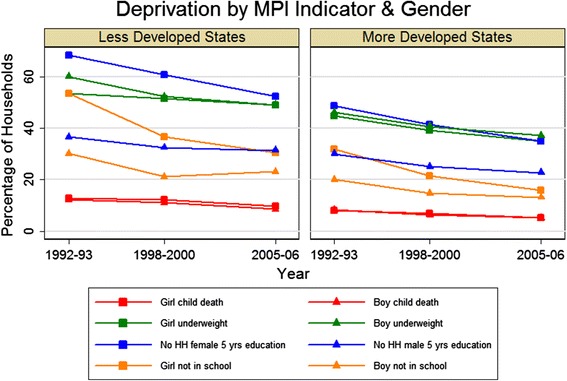
**Gender disparities within households.** Gender disparities in child death, child underweight, absence of any household member with five or more years of completed education, and any child aged 7-14 not attending school. Symbols indicate the gender for each indicator (square for females, triangle for males).

It is evident in Figure 3 that, following indicators in the standard of living dimension, the next largest deprivation was in households having an underweight child. Even in more developed states in 2005-06, in 37.4% of households with a child under age 3, one or more were underweight. Minimal progress is seen in both groups of states over time. With the exception of less developed states in 1992-93, when the proportion underweight was greater for male children, there was no clear difference in child underweight between boys and girls (Figure 5).

#### Education

The education dimension of the MPI shows a significant proportion of households in both groups of states did not have any household member with five completed years of education and had a school-age child not attending school (Figure 3). Using more recent data from the 2009-10 NSSO Employment Survey, we find 23% of households in less developed states and 15% of households in more developed states did not have any household member with five or more years of education and 14% and 7% of households had one or more school-age child not attending school, respectively.

The gender disparity was greater for households without a male/female with five years of education than for households with a boy/girl not in school (Figure 5). Between 1992-93 and 2005-06, the gap in the proportion of households without a female versus without a male with five years of education declined from 31% to 21% in less developed states and from 19% to 12% in more developed states. Across both state groups, the proportion of households with a girl child not in school decreased by over 15% between 1992-93 and 2005-06, whereas the proportion of households with a boy child not in school declined by about 7% in each group.

### Other SDH

#### Employment and financial protection

In the 2009-10 NSSO employment survey, of those respondents working in a wage or salaried position, only 20.3% in less developed states and 22.3% in more developed states reported having any form of a job contract. Only 7.7% of households in less developed states and 13.1% of households in more developed states had a household member employed in a position providing any social security benefit. Among workers, persistent disparities by industry existed across both groups of states. The vast majority of rural workers were employed in agriculture, fishing, or forestry; members of SC/ST were much more likely to work in construction; and manufacturing, trade, and education remained industries of non-SC/ST. Each of these disparities showed little to no change between 1993-94 and 2009-10.

Across four rounds of NSSO surveys from 1993-94 to 2009-10, labor force participation rates for men aged 15-59 years were steadily close to 85% in both groups of states; rates for women remained around 25% in less developed states and 35% in more developed states. Generalized progress on child labor was evident: there were steady declines in all subgroups, with the exception of SC/ST males in urban areas of less developed states. Overall, from 1993-94 to 2009-10, the proportion of children aged 5-14 years whose primary activity was working or looking for work declined from 4.2% in less developed states and 5.7% in more developed states to 1.3% in both groups of states.

#### Political participation

**Figure 6:**
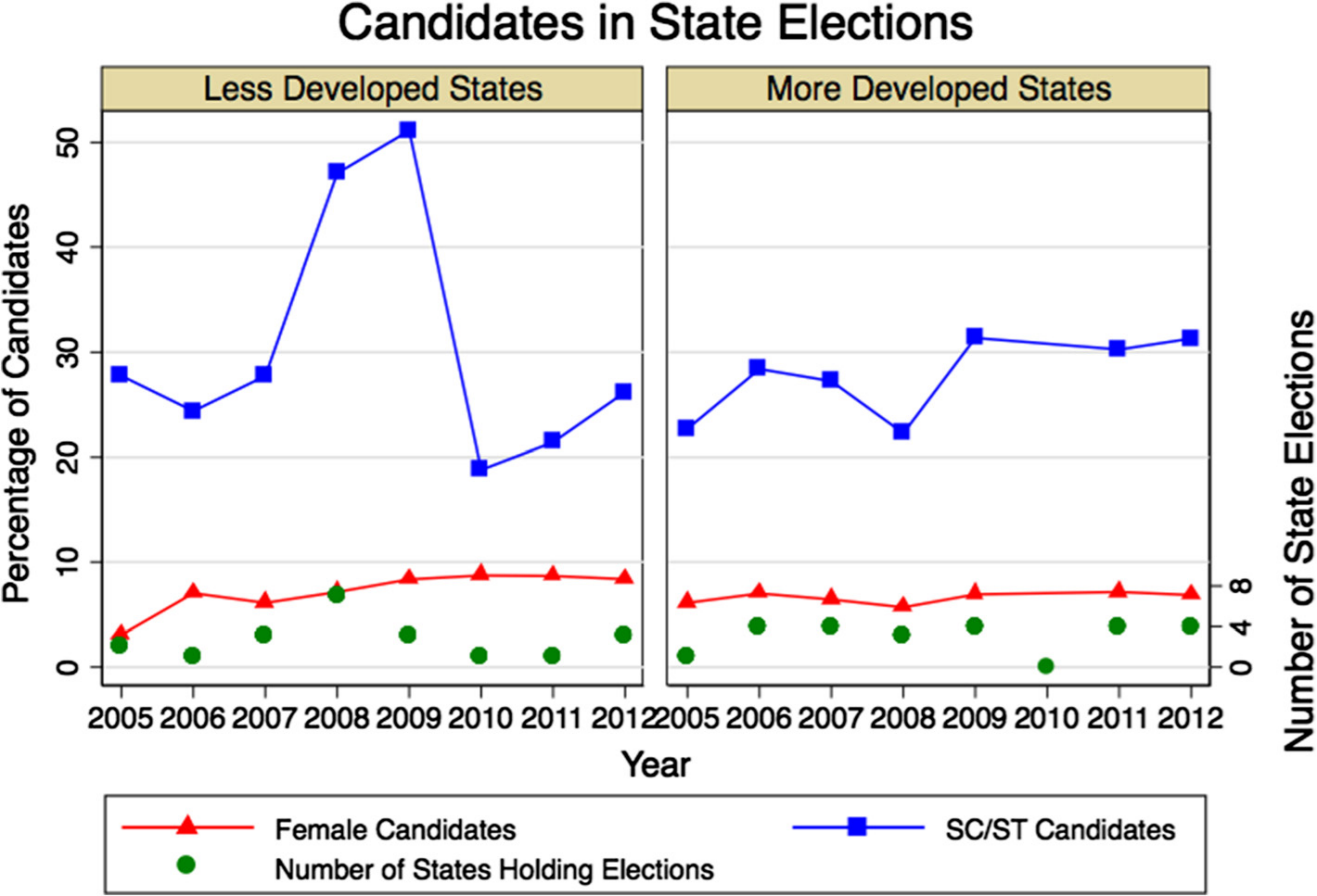
**Gender and caste proportions of candidates in state elections in India, 2005-12.** The right axis displays the number of states holding elections in each year.

Figure 6 displays data on the sex and caste of candidates in state elections held between 2005 and 2012 [20]. Fluctuations in the proportion of SC/ST candidates are attributable to the changing composition of states holding elections in each year; spikes in the less developed states are explained by a few northeast states holding elections in 2008 and 2009. These are the only states in India with majority SC/ST populations and a consistently high proportion of SC/ST candidates. Overall, nearly a third of candidates during 2005-12 belonged to SC/ST, which is proportionate to the fraction of the national population belonging to this group. In contrast, regardless of the states with elections in each year, the low percentage of female candidates showed little change and was similar in both state groups.

In all state elections, voting rates for women in less and more developed states increased from 51% and 63% in elections during 1990-94 to 64% and 78% in 2010-13, respectively [19]. Voter turnouts for men during these elections were 63% and 67% in 1990-94 and 62% and 78% in 2010-13, making the female voting rate equal to or higher than males across both groups of states in the 2010-13 elections. Caste disparities in voting rates also decreased, with deficits among SC/ST of 7% and 3% in less and more developed states in 1990-94 reversed to 1% and 2% higher rates in 2010-13 elections, respectively.

#### Environment

**Figure 7:**
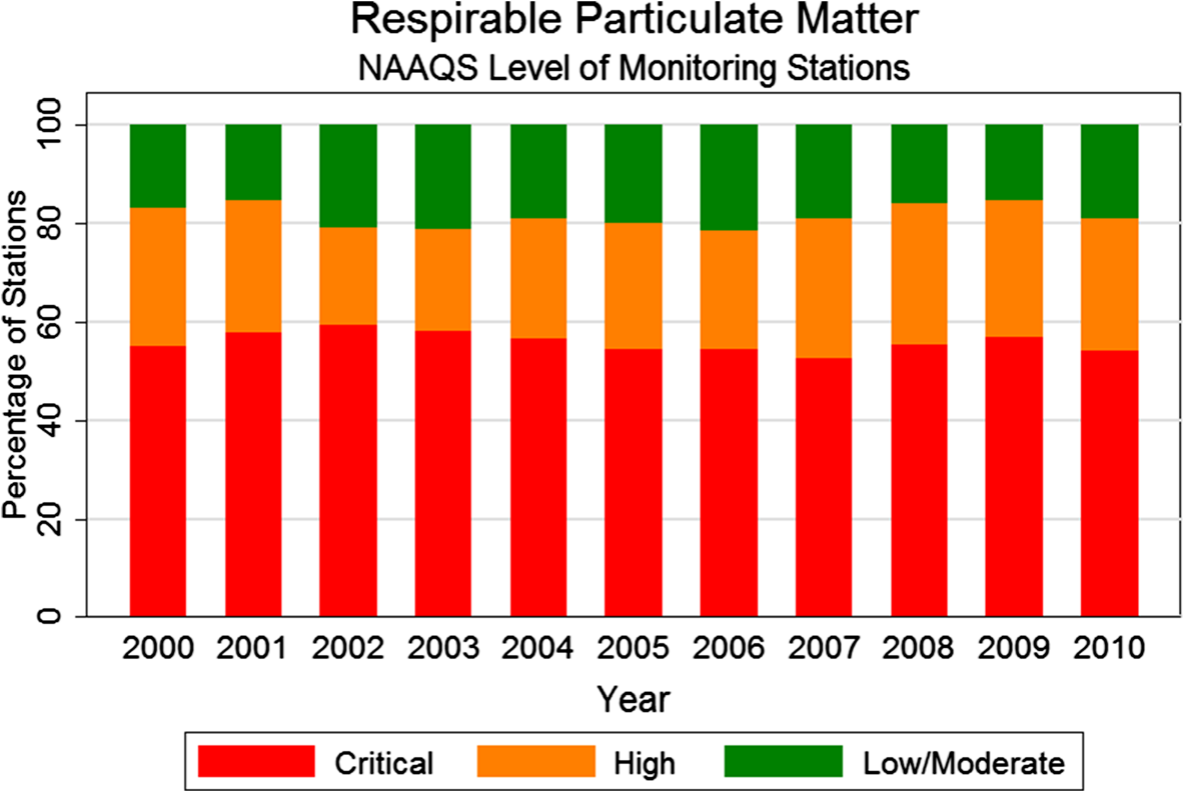
**NAAQS levels of annual mean concentrations of respirable particulate matter (PM**_**10**_ **), 2000 to 2010.** Measurements taken at air quality monitoring stations during 2000 to 2010. *NAAQS is National Ambient Air Quality Standards. Low/Moderate, High, and Critical refer to annual mean concentrations of less than 60, 60-90, and more than 90 PM_10_ micrograms/meter, respectively [23].

Between 2000 and 2010, the percentage of air quality monitoring stations with annual average concentrations of NO_2_ above the standard fluctuated between 14% and 19%; the percentage of stations above the SO_2_ threshold was consistently close to 0. Results for PM_10_ are displayed in Figure 7: 78% to 85% of stations were above the threshold every year, with more than half of the stations exceeding the standard by more than 50% (“critical” level) [21,27]. During 2011, 37% of water quality monitoring stations had a mean B.O.D. concentration higher than the general recommended threshold, and a number of rivers had concentrations in far excess of the criteria limits for both total coliform and fecal coliform [22].

## Discussion

From these analyses of trends in SDH in India over the past two decades, five issues emerge as the most urgent to address: air pollution (both indoor and outdoor), child undernutrition, unimproved sanitation, employment conditions, and gender inequality. These priorities coincide with the major risk factors contributing to lost years of healthy life in India, as identified in disease burden analyses. We discuss trends in each of these priority areas in the context of relevant national policies over the past two decades, which are summarized in Additional file 3: “Major national SDH policies.”

Household surveys reveal the striking proportion of the Indian population exposed to indoor air pollution, which is particularly significant for women and young children who typically spend more time inside near stoves. Data from air quality monitoring stations nationwide indicate dangerous levels of particulate matter in most of the country. Both household air pollution and ambient particulate matter were among the top 10 risk factors contributing disability adjusted life years (DALYs) lost in India in 1990 and in 2010 [28]. This persistence of outdoor air pollution has occurred during a period of over twenty years with no major new environmental legislation and reducing indoor air pollution has not been the focus of any national schemes. As of 2013, half of the twenty most polluted cities in the world, including the worst four, are in India [29]. Policies that provide access to cleaner fuels or improved stoves have been identified as cost-effective across a variety of national contexts [30]; and China recently demonstrated health improvements achievable through policies to reduce ambient air pollution [31], indicating possible policy approaches for India to address its indoor and outdoor air hazards.

Child undernutrition continues to affect a significant proportion of households in more and less developed states alike, and has failed to significantly decline despite national economic growth [32,33]. This is crucial in terms of the large number of children affected as well as the life-long implications for cognitive development and adult health [34]. Childhood underweight was a top five risk factor for the disease burden in India in both 1990 and 2010 [28]. Data from the 2011 national HUNGaMA Survey confirm a continued lack of progress in child undernutrition in the most recent years [35], despite this being the focus of two of the longest-running national schemes. In 1965, the Public Distribution System began to provide subsidized food to poor families [36]; and the Integrated Child Development Services Scheme started in 1975 with a focus on early childhood health and nutrition [37]. India’s continuing challenge of child undernutrition, with a high prevalence and the greatest number of undernourished children in the world [38], suggest these national programs should be evaluated and improved. A variety of effective interventions exist to address child undernutrition [39], which should be considered and, if feasible, be implemented effectively in India.

Unimproved sanitation facilities also remain too common in India, most significantly in rural areas. The prevalence of unimproved sanitation in the less developed states during the 2010-11 Annual Health Survey does not show dramatic improvement from the 2007-09 DLHS findings presented here [40]. Unimproved sanitation contributes to the spread of many infectious diseases, of which India still has a substantial burden [41]. This is in spite of almost three decades of national policies addressing this issue: the Central Rural Sanitation Programme was implemented in 1986 and most recently reconfigured as Nirmal Bharat Abhiyan in 2012 [42]. These policies should be examined for their effectiveness and potentially reconfigured using successful programs in other countries as models; much of east and southeast Asia have achieved larger gains in the percentages of their populations with access to improved sanitation over the past twenty years [43].

India has made progress in reducing child labor, but many other aspects of employment conditions continue to be problematic – a lack of job security, insufficient safety measures, and inadequate compensation. Occupational health and safety are covered in piecemeal fashion by schemes for specific sectors, but until the 2009 National Policy on Safety, Health, and Environment at Work Places, no comprehensive national policy existed, and this has yet to be fully implemented [44]. The 1987 National Child Labor Policy preceded the observed declines in child labor [45]. The ongoing implementation of the National Policy on Safety, Health and Environment at Work Places should be evaluated to assess whether it leads to improvements in occupational health and safety.

Finally, gender-based inequities persist in employment and governance, limiting women’s power in households, businesses, and private and public decision-making. At the national level, the percentage of women representatives in the *Lok Sabha*, the larger national parliament body, remained low over the past decade, from 9% in 2000 to 11% in 2012 [46]. Two recent national policies specifically target gender inequality: the Dhanalakshmi Scheme, started in 2008, offers cash payments for female births as well as for their immunizations and school enrollments [47], and the 2010 Rajiv Gandhi Scheme for Empowerment of Adolescent Girls provides skills training, supplemental food, and facilitates school enrollment for adolescent girls [48]. These policies have coincided with continued reductions in gender inequalities in education, but substantially more progress is needed to improve overall gender equality in India. In the World Economic Forum’s 2013 ranking of countries for gender equality in economics, health, education, and politics, India fell in the bottom third of 135 countries [49].

The availability of data was an important limitation of this review and influenced the priorities that could be identified; the limited information available to assess certain determinants highlight priorities for additional data collection. Strikingly high levels of dangerous air and water pollutants indicate an urgent need for better monitoring of environmental conditions. More detailed employment-related statistics, such as occupational hazards and the incidence of job-related injuries, should be routinely compiled [50]. For other important SDH not discussed here, there is a notable absence of data. Urban housing shortages are currently crudely estimated and more reliable projections are needed, particularly given the rapid pace of urbanization [51]. Transportation injuries and deaths are on the rise, but systematic data on road conditions and the enforcement of road safety laws do not exist [52]. No detailed estimates of the proportion of households without income security in case of unemployment, death, disability, or old age have been compiled. Finally, the impacts of transnational factors on the prices of health-related goods and services, climate change-related natural disasters, and agricultural yields should be assessed.

The persistence of these identified challenges in spite of relevant, and in some cases long-term, public policies, indicate that analytical studies are needed to understand the impact of interventions related to a variety of social determinants of health. A recent analysis of public expenditure in the Indian states over the past fifteen years found that increases in overall social sector expenditure, but not specifically health expenditures, were significantly associated with reductions in child mortality [53]. Another recent study indicated that a national employment scheme in India has the potential to reduce inequities in food consumption if implemented on an adequate scale [54]. More analytical studies of this kind can help to identify successful policies for improving SDH in India.

The current momentum for achieving universal health coverage in India is an important initiative, but sustained improvements in health outcomes require substantial actions on SDH in addition to expanding access to health services. Such preventive approaches are essential to controlling costs as health care coverage expands to a larger population and for reducing health inequalities and increasing healthy life expectancy for everyone in India.

## Conclusions

A systematic and continuing understanding of how SDH are evolving in India, as well as analyses of the impacts of changes in SDH on health outcomes, are essential for sustained improvements in the health of the majority of Indians. This report provides an overview of trends in major SDH in India to inform policy action and further analytical work in this area. The priorities identified in this study indicate areas where new or improved national policies are most critically needed, existing national policies that should be evaluated, and SDH for which more data collection are needed. The challenges are not uniform across the country. These analyses highlight striking inequities by geography, caste, and gender; rates for disadvantaged groups are in some cases worse now than they were for advantaged groups two decades ago. Progress across states has not been consistent and Indians living in rural areas continue to have worse indicators for all topics assessed. Caste-based inequities are also significant, with members of scheduled castes and scheduled tribes consistently worst off. Finally, gender inequalities affect women from the poorest to the richest households, but especially compound other disadvantages for women of lower castes and from rural areas. Improving SDH in India generally, and reducing these large disparities in particular, are critical for improving population health in India.

## Additional files

Additional file 1
**Adaptions to the MPI methodology.**
Additional file 2
**Correlations and regression with SDH indicators.**
Additional file 3
**Major national SDH policies.**
