# Supplementary material for: Social determinants of health in India: progress and inequities across states
Source: Int J Equity Health. 2014 Oct 8;13:88. doi: 10.1186/s12939-014-0088-0 (PMC4201685; doi:10.1186/s12939-014-0088-0)
Supplement: Additional file 1 — Adaptions to the MPI methodology. [file 12939_2014_88_MOESM1_ESM.docx]

**Social determinants of health in India: progress and inequities across states**

Krycia Cowling, Rakhi Dandona, Lalit Dandona

International Journal for Equity in Health

**ADDITIONAL FILE 1: Adaptions to the MPI methodology**

**Content**

Adaptions to the Multidimensional Poverty Index methodology to generate comparable estimates from NFHS-1, 2, and 3

Adaptations to the Multidimesional Poverty Index methodology required to generate comparable estimates from NFHS-1, 2, and 3, including the resulting impact on the number of households counted as deprived in that indicator. (The remaining two indicators – lack of electricity and absence of a household member with five or more years of education – were unchanged from the original methodology.)

| **DIMENSION** | **MPI INDICATOR** | **ALTERNATE INDICATOR USED** | **DIRECTION OF BIAS** |
| --- | --- | --- | --- |
| Standard of living | Household has no car or truck and owns at most one of: bicycle, motorbike, radio, refrigerator, television, or telephone^1^ | Household has no car or truck and owns at most one of: bicycle, motorbike, radio, refrigerator, or television | More deprived households |
| Standard of living | House has a dirt floor^2^ | Main housing material is kachha or semi-pucca | More deprived households |
| Standard of living | The drinking water source does not meet the MDG definition for “improved”, or is more than 30 minutes walk^3^ | The drinking water source does not meet the WHO/UNICEF JMP criteria for “improved” | Less deprived households |
| Standard of living | The sanitation facilities do not meet the MDG definition for “improved”, or the toilet is shared^3^ | The sanitation facilities do not meet the WHO/UNICEF JMP criteria for “improved” | Less deprived households |
| Standard of living | The household cooks with wood, charcoal, or dung^3^ | The household cooks with biomass fuels, as defined in the MDGs | More deprived households |
| Health | At least one household member is malnourished^4^ | Any child under 3 years of age, born to an ever-married woman, is malnourished | Less deprived households |
| Health | One or more children have died^5^ | Any child born to an ever-married woman in the last five years has died | Less deprived households |
| Education | At least one school-age child is not enrolled in school^6^ | Any 7-14 year old did not attend school at all during the previous year | Less deprived households |

1 - In the NFHS-1, telephone was not one of the assets included in the section on household ownership of goods. For comparability, we have excluded ownership of a telephone as criteria for this indicator across all three datasets.

2 - This indicator is the one that differs most significantly between the original MPI methodology and the one used here. Whether the household has a dirt floor can only be assessed in the NFHS-3 dataset and not in the earlier two rounds of the NFHS. In NFHS-3, dirt floor (yes/no) is constructed from the household’s main floor material, by classifying "mud/clay/earth" or "sand/dung" as “yes” for dirt floor and all other responses as “no”. Based on the relationship between this constructed variable and the main type of housing material (“dirt_floor” and “shnfhs2” in the crosstab below, respectively) in this dataset, we decided to use the indicator “main housing material is kachha or semi-pucca” as a substitute for “house has a dirt floor”.

3 - For improved drinking water, improved sanitation, and solid fuel use, we used the definitions adhered to by the relevant UN agencies as opposed to the slight variations from these definitions which are used in the original MPI methodology. This was not due to issues of data availability, but a methodological choice regarding what was most important to measure. Categories for improved drinking water and improved sanitation were taken from the WHO/UNICEF Joint Monitoring Program (JMP) (<http://www.wssinfo.org/definitions-methods/watsan-categories/>). Categories for solid fuel use were taken from MDG Goal 7 (<http://mdgs.un.org/unsd/mdg/Metadata.aspx?IndicatorId=29>).

4 - In the three rounds of the NFHS, there were variations in both the sampling frame for women selected as respondents as well as for which household members were chosen for the collection of anthropometric data, both of which impacted the comparability of the malnutrition data:

- In the NFHS-1, the sample frame was ever-married women, 15-49; anthropometric data were collected from all children under the age of four living with their mother and whose mother completed the women’s questionnaire.
- In the NFHS-2, the sample frame was ever-married women, 15-49; anthropometric data were collected from women completing the women’s questionnaire and all children under the age of three living with their mother and whose mother completed the women’s questionnaire.
- In the NFHS-3, the sample frame was all women, 15-49; anthropometric data were collected from women completing the women’s questionnaire and all children under the age of five listed in the household questionnaire.

Across the three datasets, the comparable sample for which to use the anthropometric data is all children under the age of three living with their mother, who was ever-married and completed the women’s questionnaire. As per the MPI methodology, we used a weight-for-age z-score less than two standard deviations below the median of the reference population. Weight-for-age z-scores for all children with anthropometric data are included in the NFHS datasets, however these z-scores are not comparable because in the NFHS-1 and 2, these were computed using the CDC Standard Deviation-derived Growth Reference Curves derived from the NCHS/FELS/CDC Reference Population and in the NFHS-3, the revised reference standards as recommended by the WHO Multicenter Growth Reference Study Group, 2006, were used. We manually computed the z-scores using the newer WHO reference standard for all three datasets.

5 - Because of differences in the sampling frame for female respondents - who were asked about children ever born and children who ever died - in the different survey rounds, we restricted this indicator to children of ever-married women for comparability across surveys. (NFHS-1 & 2 sampled ever-married women, 15-49; NFHS-3 sampled all women, 15-49).

6 - Alkire & Seth (2013) have a clear explanation of the comparability problems of the school attendance question. The main difference is that the NFHS-3 asked about attendance during the previous school year while NFHS-1 and 2 asked about attendance at the time of the interview, posing a comparability problem for six-year-olds who would not have been enrolled in the previous year since schooling begins at age six. For maximum comparability, we used the 7-14-year old age group.
