# Supplementary material for: Social determinants of health in India: progress and inequities across states
Source: Int J Equity Health. 2014 Oct 8;13:88. doi: 10.1186/s12939-014-0088-0 (PMC4201685; doi:10.1186/s12939-014-0088-0)
Supplement: Additional file 2 — Correlations and regression with SDH indicators. [file 12939_2014_88_MOESM2_ESM.docx]

**Social determinants of health in India: progress and inequities across states**

Krycia Cowling, Rakhi Dandona, Lalit Dandona

International Journal for Equity in Health

**ADDITIONAL FILE 2: Correlations and regression with SDH indicators**

**Content**

Multidimensional Poverty Index (MPI) correlations across and within dimensions

Logistic regressions with MPI health outcome indicators as functions of MPI SDH indicators

State-level correlations of various SDH

Multidimensional Poverty Index (MPI): Correlations by dimension

MPI Education & MPI Health = .16

MPI Education & MPI Standard of Living = .43

MPI Health & MPI Standard of Living = .25

MPI: Correlations by indicator

Indicators, in order presented: *any child death in the household, any child malnourished in the household*, *no household member with five or more years of education, any school-age child not attending school*, *unimproved source of drinking water, unimproved sanitation, indoor biomass fuel use, low quality housing, lack of electricity, and limited household asset ownership.*

Correlations of MPI health dimension indicators (health outcomes) with other MPI dimensions (social determinants of health):

Child death & MPI Education = .08

Child death & MPI Standard of living = .12

Child malnourished & MPI Education = .16

Child malnourished & MPI Standard of living = .24

Logistic regressions with MPI health dimension indicators as outcome variables and other MPI indicators as predictor variables, using household-level data:

Child death:

Child malnourished:

Correlations of various SDH at state level

Because of the different data sources used (different survey years, different states holding elections), correlations are presented for data collected within the same five-year period.

Indicators, in order presented: *MPI headcount ratio, female labor force participation rate, child labor rate, female voting rate, SC/ST voting rate, proportion of female candidates, and proportion of SC/ST candidates*.
