# Supplementary material for: Social determinants of health in India: progress and inequities across states
Source: Int J Equity Health. 2014 Oct 8;13:88. doi: 10.1186/s12939-014-0088-0 (PMC4201685; doi:10.1186/s12939-014-0088-0)
Supplement: Additional file 3 — Major national SDH policies. [file 12939_2014_88_MOESM3_ESM.docx]

**Social determinants of health in India: progress and inequities across states**

Krycia Cowling, Rakhi Dandona, Lalit Dandona

International Journal for Equity in Health

**ADDITIONAL FILE 3: Major national SDH policies**

**Content**

Major national policies addressing social determinants of health in India, active from 1990 to present

Major national policies active since 1990 which address social determinants of health in India, by primary SDH domain addressed and year of implementation. Policies without an end date are ongoing.

| **POLICY** | **YEARS** | **DESCRIPTION** |
| --- | --- | --- |
| **Living Conditions** | | |
| Central Rural Sanitation Programme | 1986-1999 | Improve sanitation in rural areas, with a focus on the needs of women |
| Indira Awaas Yojana | 1999 | Subsidies to rural families for housing construction |
| Jawahar Gram Samridhi Yojna | 1999 | Community village infrastructure and supplementary employment for unemployed poor in rural areas |
| Total Sanitation Campaign | 1999-2012 | Increase awareness and generate demand for improved sanitation in rural areas |
| Valmiki Ambedkar Aawas Yojan | 2001 | Targeted urban housing construction for slum dwellers, including community toilets |
| Bharat Nirman | 2005 | Rural infrastructure projects: safe drinking water, roads, homes, electricity, telephone lines, irrigation |
| Jawaharlal Nehru National Urban Renewal Mission | 2005 | Developing urban infrastructure and providing basic services to the urban poor |
| National Urban Sanitation Policy | 2008 | Reducing open defecation and improving sanitation in urban areas |
| Multisectoral Development Programme | 2008 | Infrastructure development in districts with high proportions of minority populations |
| National Water Policy | 2012 | Improving access to clean drinking water and protecting and managing water resources |
| Nirmal Bharat Abhiyan | 2012 | Building and improving sanitation facilities in rural areas |
| **Undernutrition** | | |
| Public Distribution System | 1965 | Provision of subsidized wheat, rice, kerosene, and sugar |
| Integrated Child Development Scheme | 1975 | Provision of food, health services, and health education to children below six years of age and pregnant and lactating women |
| Mid-Day Meal Scheme | 1995 | Free meals for children in grades 1-8 in government and aided schools. |
| **Education** | | |
| Sarva Shiksha Abhiyan | 2001 | Free and compulsory education for all children ages 6-14 |
| Rashtriya Madhyamik Shiksha Abhiyan | 2009 | Improving access to and quality of secondary education |
| Right of Children to Free and Compulsory Education Act | 2009 | Free and compulsory education for all children ages 6-14 |
| Saakshar Bharat Programme | 2009 | Promoting and strengthening adult education, especially for women |
| **Gender inequality** | | |
| 73rd Amendment Act | 1992 | Reservation of one-third of all seats in *Panchayat Raj* Institutions for women |
| Dhanalakshmi Scheme | 2008 | Cash payments for birth of girl child and subsequently, for immunizations completed and school enrollments at each level |
| Rajiv Gandhi Scheme for Empowerment of Adolescent Girls | 2010 | Training for domestic and vocation skills, provision of grains, and facilitating school enrollment for adolescent girls |
| **Employment** | | |
| National Child Labor Policy | 1987 | Reduce child labor and rehabilitate child workers |
| [Swarna Jayanti Shahari Rozgar Yojana](http://en.wikipedia.org/wiki/Swarna_Jayanti_Shahari_Rozgar_Yojana) | 1997 | Employment for unemployed and underemployed urban poor |
| Jawahar Gram Samridhi Yojna | 1999 | Community village infrastructure and supplementary employment for unemployed poor in rural areas |
| Swarnjayanti Gram Swarozgar Yojana | 1999 | Development of self-employment for rural poor |
| Mahatma Gandhi National Rural Employment Guarantee Scheme | 2006 | Guaranteed employment in unskilled and low skilled public work for rural households |
| National Policy on Safety, Health, and Environment at Work Places | 2009 | Reduce work-related injuries and improve awareness of monitoring of occupational health and safety issues |
| **Environment** | | |
| The Water (Prevention and Control of Pollution) Act | 1974 | Prevention and control of water pollution, maintaining and restoring water quality, and the establishment of a water pollution board |
| The Air (Prevention and Control of Pollution) Act | 1981 | Prevention, control and abatement of air pollution in India |
| Environment (Protection) Act | 1986 | Empowers the Central Government to establish authorities to prevent environmental pollution in all its forms and to tackle specific environmental problems |
| **Financial protection** | | |
| National Social Assistance Scheme | 1995 | Financial assistance to elderly, widowed, disabled, and unemployed persons; food assistance to elderly |
| Rashtriya Swasthya Bima Yojana | 2008 | Health insurance for poor families and informal sector workers |
